# Potential of Fasting C-peptide to Glucose Ratio and Triglyceride Glucose Index as Markers for β-Cell Dysfunction and Insulin Resistance in Patients With Type 2 Diabetes on Insulin Therapy

**DOI:** 10.7759/cureus.66543

**Published:** 2024-08-09

**Authors:** Breshan S Essa, Mohammed Q Meena

**Affiliations:** 1 Endocrinology and Diabetes, Ninawa Health Directorate, Mosul, IRQ; 2 Medicine, College of Medicine, Hawler Medical University, Erbil, IRQ

**Keywords:** type 2 diabetes mellitus, insulin therapy, β-cell dysfunction, insulin resistance, c-peptide, triglyceride

## Abstract

Background: Pathogenesis of type 2 diabetes mellitus (T2DM) is combined from initial insulin resistance (IR) and subsequent β-cell dysfunction. Insulin therapy can replace β-cell function in advanced stages. However excessive insulin therapy increases IR and may expose the patients to risk of cardiovascular disease. We aim to assess β-cell function and IR in patients with type 2 diabetes on insulin therapy by fasting C-peptide to glucose ratio (FCPGR), and triglyceride glucose (TyG) index respectively to support treatment plans.

Method: A cross-sectional study was conducted at the Galiawa Diabetes and Endocrinology Teaching Center in Erbil City, Iraq, from June 2023 to January 2024. A convenient sample of 100 patients with T2DM on insulin-based therapy were included after obtaining informed written consent and excluding conditions such as acute illness, uncertain type of diabetes, etc. Each patient was evaluated for anthropometric parameters and current treatment details. Biochemical tests were then carried out to calculate metabolic syndrome (MetS) index score, FCPGR, and TyG index. Finally, patients were divided into four subgroups according to their FCPGR and TyG index and the data were analyzed statistically.

Result: The data showed those patients with sufficient β-cell function were 60 (60%), and patients with high TyG index were 95 (95%). There was a significant negative correlation between FCPGR and hemoglobin A1c (HbA1c) (p-value=0.001), while there was a positive correlation between TyG index and HbA1C (p-value=0.001). None of these markers were correlated with BMI (p-value=0.297, and 0.976), duration of T2DM (p-value=0.258, and 0.458), and dose of insulin therapy (p-value=0.901, and 0.477). Patients with sufficient β-cell function and high TyG index had the lowest HbA1C.

Conclusion: The study provides valuable insights into the utility of FCPGR and TyG index as biomarkers for β-cell function and insulin resistance in T2DM patients on insulin therapy. The significant correlation with HbA1C underscores their potential in clinical practice. However, the lack of correlation with BMI, disease duration, and insulin dose suggests that further investigation is needed to fully understand these biomarkers' implications across diverse patient profiles.

## Introduction

Type 2 diabetes mellitus (T2DM) is a chronic progressive disease in which insulin resistance (IR) leads to compensatory hyperinsulinemia with subsequent β-cell exhaustion, ultimately ending with insulin deficiency [1]. Insulin therapy in T2DM is indicated when the β-cell function no longer copes with the body's insulin requirement to control hyperglycemia. Timely initiation of insulin therapy achieves euglycemia within a short period, regenerates residual β-cells, and prevents rapid decline in β-cell function [2]. On the other hand, over-treatment with insulin can result in weight gain, IR, risk of hypoglycemia, and cardiovascular and/or kidney-related morbidity and mortality in those who have already IR [3]. Conversely, inappropriate delay of insulin therapy may cause glucolipotoxicity and diabetes-related complications [4].

The initial decision to start insulin therapy is usually based upon clinical guideline recommendations and other patient-related factors including body mass index (BMI), age, duration of T2DM, complications of T2DM, and target glycated haemoglobin (HbA1c). However, in cases where patients started empirical insulin therapy, the decision to continue or change to non-insulin therapy during follow-up is not addressed by the guidelines. Assessment of residual β-cell function can guide the proper timing of insulin therapy commencement in patients with T2DM [5]. There are many biochemical markers advocated to assess β-cells function. C-peptide is one of these markers that is widely accepted for assessing β-cells functional reserve. C-peptide is a 31-amino acid chain, detached from proinsulin and secreted in equimolar amounts to insulin. C-peptide does not undergo hepatic first-pass metabolism, has a longer half-life than insulin (20-30 minutes versus 3-5 minutes) and its measurement is not affected by exogenous insulin therapy. Altogether this makes C-peptide a better marker for assessing β-cell function than insulin in patients on insulin therapy. Among different methods of C-peptide estimation, plasma fasting C-peptide is convenient, less time-consuming, and unaffected by food composition [6]. However, hyperglycemia stimulates C-peptide release, so the C-peptide level should be corrected to the degree of hyperglycemia. Fasting C-peptide to glucose ratio (FCPGR) was evaluated to have a good correlation with β-cell function [5].

Insulin secretion and the C-peptide also rise in response to increased IR in those with preserved β-cell function [7]. Targeting IR in the management plan unloads β-cells and restores some of the imbalance between insulin synthesis and requirement.

Triglyceride-glucose (TyG) index is a well-established, simple, and inexpensive marker of IR. TyG index utilizes triglyceride (TG) and glucose levels in its calculation [8]. IR causes hypertriglyceridemia by increasing lipolysis in the adipose tissue, overproduction of TG-rich lipoproteins in the liver, and decreasing lipoprotein lipase (the enzyme responsible for the removal of chylomicron-rich TG from the circulation) [9].

This study aims to examine the potential use of FCPGR and TyG index as markers for β-cell function and IR respectively in patients with T2DM who are on insulin therapy.

## Materials and methods

Study design

A cross-sectional study was conducted in the Galiawa Diabetes and Endocrinology Teaching Center of Erbil City, Iraq, from June 2023 to January 2024. The study was approved by the Ethical Committee of Faiha Specialized Diabetes, Endocrine, and Metabolism Center (FDEMC) (ref #56/35/28) on March 19th, 2023, and conformed to the 1964 Declaration of Helsinki and its later amendments or comparable ethical standards.

Inclusion criteria

We recruited patients who were registered at the Galiawa Diabetes and Endocrinology Teaching Center with T2DM during their routine follow-up visit and who were already on insulin therapy, either as once or multiple daily insulin injections, as monotherapy or in combination with oral antidiabetic agents, for at least three months. Each patient was included only if they provided informed written consent. After excluding those who met exclusion criteria, a total of 100 patients were included.

Exclusion criteria

We excluded any patient with uncertain type of diabetes, acute dysglycemia, serum glucose level <70 mg/dl, pregnancy, patients on drugs known to cause hyperglycemia (e.g., steroids), renal transplant, chronic kidney disease with creatinine clearance <50 ml/min, advanced liver disease, critically ill, hemoglobinopathy, TG >500 mg/dl, pancreatitis (acute or chronic), hypothyroidism, alcohol intake or malignancy.

Data collection

All patients were already diagnosed according to the American Diabetes Association (ADA) diagnostic criteria for diabetes and the type of diabetes was further identified by careful history and clinical examination. The age, gender, and details of diabetes treatment were retrieved from the patients’ records. The duration of T2DM was calculated from the time of first diagnosis that was recalled by the patient and/or their relatives. The score of metabolic syndrome (MetS) index followed the Adult Treatment Panel III 2005 criteria. The criteria include the presence of diabetes mellitus, waist circumference (WC) ≥102 cm for men and ≥88 cm for women, hypertriglyceridemia (≥150 mg/dL or on treatment for elevated TG), low high-density lipoprotein (HDL) cholesterolemia (HDL level of <40 mg/dL for men and <50 mg/dL for women or on treatment for reduced HDL), high blood pressure (BP) (systolic BP of ≥130 mmHg, or diastolic BP of ≥85 mmHg, or on antihypertensive medication) in which the lowest score is 1, and the highest score is 5. The MetS index score of 3 or more defines the presence of MetS [10].

The anthropometric measurements were assessed by the chief researcher for all patients who were fasting. The weight and height of each patient were measured wearing light clothes and shoes off, then the BMI was calculated using the equation: $weight (kg)/ height(m^{2})$. The World Health Organization classification for the non-Asian population was used to define underweight (BMI value <18.5 Kg/m2), normal weight (BMI between 18.5-24.9 Kg/m2), overweight (BMI between 25-29.9 Kg/m2), and obese (BMI ≥30 Kg/m2). Obesity is further sub-classified into class I (in which BMI is between 30 - 34.9 Kg/m2), class II (BMI between 35 - 39.9 Kg/m2), and class III (BMI ≥ 40 Kg/m2) [11]. The WC was measured by flexible plastic tape horizontally just above the iliac crest in standing position at the end of expiration according to the Centers for Disease Control and Prevention recommendations. The BP was measured using a mercury sphygmomanometer rather than an automated device to avoid measurement error in those who have arrythmia. The BP was measured from both patients’ arms after sitting on a chair for at least five minutes and the highest measurement was recorded depending on Korotkoff Phase I (for systolic BP) and Phase V (for diastolic BP).

Biochemical tests

All recruited patients were fasting for ≥12 hours when peripheral venous blood samples were withdrawn. Furthermore, they did not take their morning dose of insulin or antidiabetic agents. For each patient, withdrawn blood was transferred into a purple tube containing EDTA and a yellow tube containing a separation gel and coagulant which were labelled properly. Serum was separated immediately after centrifugation and laboratory biochemical tests were performed within three to five hours using electrochemiluminescence immunoassay kits. Roche Cobas c311 analyzer (Roche, Basel, Switzerland) was used to measure the HbA1c, fasting serum glucose (FSG), fasting TG, and HDL cholesterol concentrations. Roche Cobas e411 analyzer was used to measure serum C-peptide, with sensitivity to detect C-peptide levels as low as 0.01 ng/ml.

The FCPGR was calculated by the following equation [5]:

$Fasting C-peptide (ng/ml)/FSG (mg/dl)\times 100$

In which FCPGR <0.87 is defined as insufficient β-cell function and FCPGR ≥0.87 is defined as sufficient β-cell function [5].

The TyG index was calculated by the following formula [12]:

$Ln [Fasting TG (mg/dl) \times FSG (mg/dl)]/2$

In which TyG index of >4.68 [8] is defined as a high TyG index, and ≤4.68 as a low TyG index.

Statistical analysis

All data were calculated and analyzed using the statistical package of social science (SPSS) software, version 26 (IBM Corp., Armonk, NY, USA). Descriptive statistics were used to present the recruited patients' characteristics as mean, standard deviation (SD), and percentage (%). Kolmogorov-Smirnov test was used to test the normality of data distribution. Kruskal Wallis test and Mann-Whitney test were used to find out the difference in the mean of FCPGR and TyG index among the categorical groups, while the Spearman correlation test was used to determine the relationship between (FCPGR, and TyG index) and the continuous study variables. Thereafter, patients were classified according to cut-off points of FCPGR and TyG index into four groups and inferential tests were used to find out the difference among patients’ variables.

## Results

One hundred T2DM patients participated in this study. The mean age of the participants was 61±8.33 years. About 74% of the patients (N=74) were women. The mean duration of diabetes among patients was 16±6.52 years. Regarding the anthropometric measures, the mean WC of the patients was 106±9.67 cm. Furthermore, 81 (81%) were classified as obese. The majority (82, 82%) of the participants were hypertensive. About the lipid profile of the patients, the mean HDL level was 43.43±10.91 mg/dl while that of TG was 175.13±88.72 mg/dl. About 89 (89%) of patients had MetS. The glycemic measurements included the FSG and HbA1C with means 214.43±89.95 mg/dl and 9.44±1.83 respectively. On the other hand, the mean fasting C-peptide was 2.19±1.18 ng/ml, and the mean FCPGR was 1.11±0.61 ng/dl, while the mean TyG index was 5.17±0.32. About two-thirds (69, 69%) of the patients were on insulin therapy combined with oral anti-diabetic agents. Among those patients who were on combination therapy, metformin was the commonest agent followed by sulfonylureas (SU), dipeptidyl peptidase 4 inhibitors (DPP4i), sodium-glucose cotransporter-2 inhibitors (SGLT2i), and pioglitazone (65 (94%), 19 (27.5%), 11 (15.9%), seven (10.1%), and three (4.3%) respectively). The mean total insulin dose used by the patients was 0.67±0.36 units/kg (Table 1).

**Table 1:** General characteristics of the study participants * Percent from number of those on combination therapy BMI: body mass index; BP: blood pressure; DPP4i: dipeptidyl peptidase 4 inhibitor; FCPGR: fasting C-peptide to glucose ratio; FSG: fasting serum glucose; HbA1C: glycated haemoglobin; HDL: high density lipoprotein cholesterol; MetS: metabolic syndrome; N: number; SD: standard deviation; SGLT2i: sodium-glucose cotransporter-2 inhibitor; SU: sulfonylurea; TG: triglyceride; TyG index: triglyceride glucose index; WC: waist circumference.

Variable	
Age (years), Mean ± SD	60.75 ± 8.33
Men, N (%)	26 (26)
Women, N (%)	74 (74)
Duration of T2DM (years), Mean ± SD	15.83 ± 6.52
WC (cm), Mean ± SD	106.17 ± 9.67
Normal BMI, N (%)	6 (6)
Overweight, N (%)	13 (13)
Obesity – Class I, N (%)	45 (45)
Obesity – Class II, N (%)	26 (26)
Obesity – Class III, N (%)	10 (10)
MetS, N (%)	89 (89)
MetS index, Mean ± SD	3.91 ± 0.94
Hypertension, N (%)	82 (82)
Systolic BP (mmHg), Mean ± SD	144.7 ± 23.81
Diastolic BP (mmHg), Mean ± SD	86.40 ± 12.24
HDL (mg/dl), Mean ± SD	43.43 ± 10.91
TG (mg/dl), Mean ± SD	175.13 ± 88.72
FSG (mg/dl), Mean ± SD	214.43 ± 89.95
HbA1C (%), Mean ± SD	9.44 ± 1.83
C-peptide (ng/ml), Mean ± SD	2.19 ± 1.18
FCPGR, Mean ± SD	1.11 ± 0.61
TyG index, Mean ± SD	5.17 ± 0.32
On insulin therapy alone, N (%)	31 (31)
On combination therapy, N (%)	69 (69)
Metformin, N (%)	65 (94)*
Pioglitazone, N (%)	3 (4.3)*
SU, N (%)	19 (27.5)*
DPP4i, N (%)	11 (15.9)*
SGLT2i, N (%)	7 (10.1)*
Total insulin dose (unit/kg), Mean ± SD	0.67 ± 0.36
Basal insulin dose (unit/kg), Mean ± SD	0.43 ± 0.26
Duration of insulin therapy (years), Mean ± SD	5.68 ± 5.3

Regarding the mean of FCPGR among categorical variables, a statistically significant difference was found in the mean of FCPGR between males and females (p-value=0.029) in which male patients had higher mean FCPGR levels. Also, there was a significant difference in mean FCPGR among hypertensive and normotensive patients, with higher FCPGR among normotensives, while no statistically significant differences were found between BMI and treatment regimens (Table 2).

**Table 2:** Difference in mean FCPGR among categorical variables ¶ Kruskal Wallis test; *Mann Whitney test; ** significant p-value (≤ 0.05) DPP4i: dipeptidyl peptidase 4 inhibitor; SD: standard deviation; SGLT2i: sodium-glucose cotransporter-2 inhibitor; SU: sulfonylurea.

Variable		Mean FCPGR ± SD	P-value
Gender*	Men	1.38 ± 0.75	0.029**
	Women	1.02 ± 0.53	
BMI¶	Normal	0.67 ± 0.32	0.297
	Overweight	1.13 ± 0.51	
	Obesity - Class I	1.14 ± 0.66	
	Obesity - Class II	1.10 ± 0.60	
	Obesity - Class III	1.30 ± 0.58	
Hypertension*	Present	1.11 ± 0.59	0.028**
	Absent	1.16 ± 0.73	
Treatment*	Insulin alone	1.02 ± 0.67	0.154
	Combination therapy	1.10 ± 0.53	
Metformin*	Treated	1.10 ± 0.53	0.764
	Untreated	1.14 ± 0.74	
Pioglitazone*	Treated	1.05 ± 0.30	0.849
	Untreated	1.12 ± 0.62	
SU*	Treated	1.37 ± 0.73	0.074
	Untreated	1.05 ± 0.56	
DPP4i*	Treated	1.02 ± 0.39	0.925
	Untreated	1.13 ± 0.63	
SGLT2i*	Treated	0.98 ± 0.54	0.539
	Untreated	1.12 ± 0.61	

The study results revealed a statistically significant negative correlation between FCPGR and HbA1C (Table 3).

**Table 3:** Correlation of fasting C-peptide to glucose ratio (FCPGR) with continuous variables Spearman correlation ** significant p-value (≤ 0.05) HbA1C: glycated haemoglobin; MetS: metabolic syndrome; TyG index: triglyceride glucose index.

Variable	RhO	P-value
Age	0.078	0.439
Duration of T2DM	-0.114	0.258
MetS index	0.186	0.063
HbA1C	-0.433	0.001**
TyG index	-0.152	0.131
Duration of insulin therapy	-0.038	0.711
Total insulin dose	0.013	0.901
Basal insulin dose	0.058	0.570

Considering the TyG index, the study results found a significant difference in mean TyG index among hypertensive and normotensive patients (p<0.001) with higher mean TyG index among hypertensives. A non-significant difference in the mean of the TyG index was found between other categorical groups (Table 4).

**Table 4:** Difference in mean triglyceride glucose index among categorical groups ¶ Kruskal Wallis test; *Mann Whitney test; ** significant p-value (≤ 0.05) DPP4i: dipeptidyle peptidase 4 inhibitor; SD: standard deviation; SGLT2i: sodium-glucose cotransporter-2 inhibitor; SU: sulfonylurea.

	Variable	Mean TyG index ± SD	P-value
Gender*	Men	5.18 ± 0.35	0.984
	Women	5.16 ± 0.31	
BMI¶	Normal	5.24 ± 0.59	0.976
	Overweight	5.19 ± 0.28	
	Obesity - Class I	5.18 ± 0.33	
	Obesity - Class II	5.13 ± 0.24	
	Obesity - Class III	5.14 ± 0.32	
Hypertension*	Present	5.18 ± 0.3	0.001**
	Absent	5.13 ± 0.43	
Treatment*	Insulin therapy alone	5.21 ± 0.33	0.597
	Combination therapy	5.15 ± 0.31	
Metformin*	Treated	5.16 ± 0.30	0.945
	Untreated	5.18 ± 0.35	
Pioglitazone*	Treated	4.86 ± 0.41	0.154
	Untreated	5.18 ± 0.31	
SU*	Treated	5.13 ± 0.32	0.589
	Untreated	5.18 ± 0.32	
DPP4i*	Treated	5.17 ± 0.38	0.912
	Untreated	5.17 ± 0.31	
SGLT2i*	Treated	5.12 ± 0.54	0.898
	Untreated	5.17 ± 0.30	

Spearman’s correlation test revealed a significant negative correlation between the TyG index and age, while significant positive correlations were found between the TyG index and both the MetS index and HbA1C (Table 5).

**Table 5:** Correlation between TyG index and continuous variables Spearman correlation ** significant p-value (≤ 0.05) FCPGR: fasting C-peptide to glucose ratio; HbA1C: glycated haemoglobin; HDL: high density lipoprotein cholesterol; MetS index: metabolic syndrome index; TyG index: triglyceride glucose index.

Variable	RhO	P-value
Age	-0.216	0.031**
Duration of T2DM	-0.075	0.458
MetS index	0.375	0.001**
HDL	-0.164	0.102
HbA1C	0.407	0.001**
FCPGR	-0.152	0.131
Duration of insulin therapy	-0.047	0.639
Total insulin dose	0.072	0.477
Basal insulin dose	-0.022	0.832

**Figure 1:**
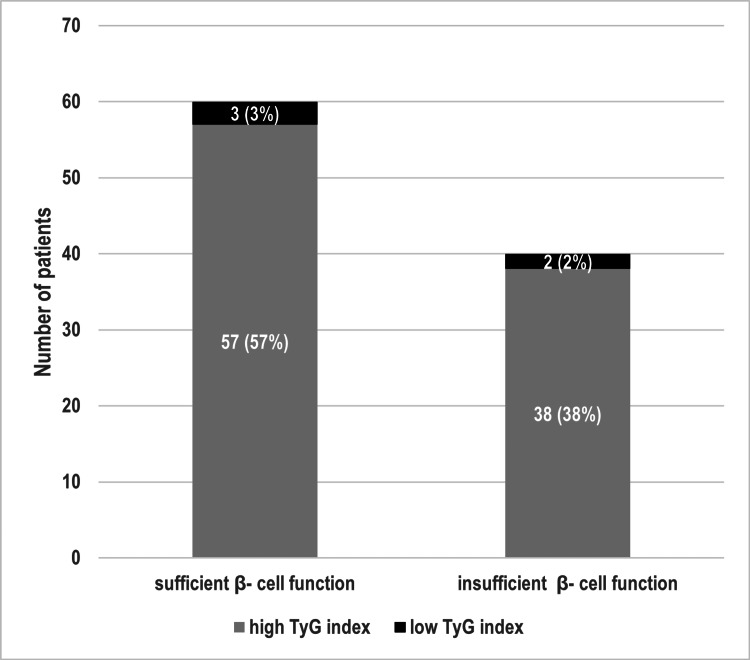
Distribution of study subgroups TyG: Triglyceride glucose index.

When the data was further subdivided according to both FCPGR and TyG index, patients with sufficient β-cell function were 60 (60%), and patients with high TyG index were 95 (95%) (Figure 1).

Finally, the Fisher exact test was used to explore the association between the subgroups and the categorical variables which revealed a significantly lower TyG index among those on combined SGLT2i or pioglitazone with insulin. Also, the Kruskal Wallis test showed significantly lower mean of HbA1C among patients with higher FCPGR and lower TyG index (Table 6).

**Table 6:** Characteristics of study subgroups ¶ Fisher exact test (number of patients); * Kruskal Wallis test (mean ±SD of variable); ** significant p-value (≤ 0.05) DPP4i: dipeptidyl peptidase 4 inhibitor; HbA1C: glycated haemoglobin; MetS index: metabolic syndrome index; SGLT2i: sodium-glucose cotransporter-2 inhibitor; SU: sulfonylurea.

Variable		Sufficient β-cell function / high TyG index (N= 57 (57%)) N (%) or mean ± SD	Sufficient β-cell function / low TyG index (N= 3 (3%)) N (%) or mean ± SD	Insufficient β-cell function / high TyG index (N= 38 (38%)) N (%) or mean ± SD	Insufficient β-cell function / low TyG index (N= 2 (2%)) N (%) or mean ± SD	P-value
Gender¶	Men	19 (19)	1 (1)	5 (5)	1 (1)	0.137
	Women	38 (38)	2 (2)	33 (33)	1 (1)	
BMI¶	Normal	2 (2)	0 (0)	3 (3)	1 (1)	0.317
	Overweight	9 (9)	0 (0)	4 (4)	0 (0)	
	Obesity - Class I	25 (25)	2 (2)	17 (17)	1 (1)	
	Obesity - Class II	14 (14)	0 (0)	12 (12)	0 (0)	
	Obesity - Class III	7 (7)	1 (1)	2 (2)	0 (0)	
Hypertension¶	Present	2 (2)	48 (48)	1 (1)	31 (31)	0.560
	Absent	1 (1)	9 (9)	1 (1)	7 (7)	
Treatment¶	Insulin alone	16 (16)	0 (0)	14 (14)	1 (1)	0.472
	Combination therapy	41 (41)	3 (3)	24 (14)	1 (1)	
Metformin¶	Treated	38 (38)	2 (2)	24 (24)	1 (1)	0.955
	Untreated	19 (19)	1 (1)	14 (14)	1 (1)	
Pioglitazone¶	Treated	2 (2)	0 (0)	0 (0)	1 (1)	0.001**
	Untreated	55 (55)	3 (3)	38 (38)	1 (1)	
SU¶	Treated	13 (13)	1 (1)	5 (5)	0 (0)	0.522
	Untreated	44 (44)	2 (2)	33 (33)	2 (2)	
DPP4i¶	Treated	6 (6)	0 (0)	4 (4)	1 (1)	0.321
	Untreated	51 (51)	3 (3)	34 (34)	1 (1)	
SGLT2i¶	Treated	2 (2)	1 (1)	3 (3)	1 (1)	0.019**
	Untreated	55 (55)	2 (2)	35 (35)	1 (1)	
Age (years)*		61.59 ± 7.90	65.66 ± 6.42	58.9 ± 9.07	63 ± 2.82	0.284
MetS index*		4.03 ± 0.96	3.66 ± 0.57	3.78 ± 0.90	3.00 ± 1.41	0.28
Duration of T2DM (years)*		15.44 ± 6.29	19 ± 6.92	16.05 ± 7.05	18 ± 2.82	0.819
Duration of insulin therapy (years)*		5.3 ± 5.11	8.5 ± 7.86	6 ± 5.70	7 ± 1.41	0.673
HbA1C (%)*		9.04 ± 1.62	6.4 ± 1.03	10.32 ± 1.71	8.65 ± 2.75	0.001**
Total insulin dose (unit/kg)*		0.65 ± 0.31	0.66 ± 0.14	0.72 ± 0.45	0.53 ± 0.24	0.930
Basal insulin dose (unit/kg)*		0.40 ± 0.209	0.45 ± 0.104	0.46 ± 0.333	0.36 ± 0.007	0.794

## Discussion

The results of this study showed that most of the recruited patients were obese, had MetS, and had poor glycemic control despite using almost the maximum basal insulin dose of 0.43 (±0.26) unit/kg/day recommended by the ADA 2024 guideline. This finding raised the concern of mismatched patients’ pathophysiological status (degree of β-cell dysfunction and IR) with the provided treatment (insulin). This concern was investigated using FCPGR and TyG index to get a better insight into current patients’ status.

The relative majority (60, 60%) of the recruited patients had sufficient β-cell function reflected by the high FCPGR values (>0.87). However, fasting C-peptide measurement cannot detect subtle β-cell function and those with FCPGR below 0.87 should be further evaluated by glucagon stimulation test or mixed meal stimulation test before considering them as absolute insulin-deficient patients [6]. Therefore, FCPGR is a specific but not sensitive marker [5] to detect preserved β-cell function, making it a suitable initial test to aid the decision to change treatment from insulin to non-insulin therapy.

Sufficient β-cell function subgroups have significantly lower HbA1C than those with insufficient β-cell function subgroups which is in agreement with previous studies [13,14]. The FCPGR significantly negatively correlated with HbA1C. These findings support the use of FCPGR as an indicator of β-cell function since good glycemic control in the absence of significant difference among the subgroups regarding other variables (age, BMI, duration of T2DM, MetS index score, duration on insulin therapy, insulin dosing, or being combined with oral antidiabetic agents) would reflect endogenous β-cell reserve.

The non-significant correlation of FCPGR with age, BMI, and dose of insulin was in agreement with the finding of Iwao et al. (2012) [15] in which there was no significant difference among patients with successfully (higher C-peptide indices) switched to liraglutide and unsuccessful (lower C-peptide indices) patients regarding the former mentioned variables. Similarly, Scionti et al. (1992) [16] found that normal BMI does not exclude presence of preserved β-cell function and successful transition to non-insulin therapy is still possible.

The finding of this study showed a non-significant correlation between FCPGR and the duration of T2DM. This finding was not in line with Iwao et al.'s 2012 study findings [15], which found significantly higher C-peptide indices and shorter duration of T2DM in those who successfully transitioned to non-insulin therapy. This could be attributed to recall bias since this information was collected from the patients or their relatives, because many patients had T2DM diagnoses a long time before their registration in the Galiawa Diabetes and Endocrinology Teaching Center.

The results of this study also suggest that men are more likely to have sufficient β-cell function compared to women. This finding could be attributed to different factors including hormonal/gestational history and body fat composition among women, in addition to educational and psychosocial issues [17].

The results of this study also found a statistically insignificant association between β-cell function and different treatment regimens, although it is closer to significance in regard to SU treatment (p-value 0.07). SU stimulates β-cells and results in an expected higher level of C-peptide, however its effect on β-cells diminishes over time since it is β-cell reserve dependent. Furthermore, assessment of β-cell function also can be used as a guide for discontinuation of SU treatment [18]. Non-significant difference between the mean of FCPGR among patients SU treated and those SU untreated can be explained by overlap of FCPGR level among already sufficient β-cell function and those stimulated by SU, in addition to diminished effect of SU in those with insufficient β-cell function. Further prospective studies are still needed to explain this result.

Of the recruited patients, about 95 (95%) had high TyG index, 89 (89%) had metabolic syndrome and 81 (81%) were obese; these findings reflect high IR among study patients. Patients with T2DM are commonly associated with adiposity and MetS, conditions that are closely linked to IR [1]. The TyG index provides a fuller understanding of the patient's metabolic state, beyond what is offered by traditional markers like HbA1c or BMI. Furthermore, the TyG index has predictive value for fat loss induced by dietary interventions, suggesting its utility in monitoring the effectiveness of lifestyle modifications in managing T2DM [19].

A statistically significant positive correlation was found between TyG index and MetS index score. This finding strengthens the ability of TyG index to assess IR in T2DM on insulin therapy and also it is in line with Son et al. (2021), in which TyG index was the best predictive marker for MetS [20].

Against literatures’ findings, a negative statistically significant correlation was found between TyG index and age; this finding could be affected by multiple factors including lifestyle/physical activity and degree of glycemic control [21]. More standardized research is needed to clarify this finding.

High TyG index was found among all BMI categories resulting in insignificant association of TyG index with BMI. This is probably due to the presence of normal-weight metabolically obese patients who have high IR despite having normal BMI [22].

The TyG index was significantly associated and positively correlated with HbA1C levels. This finding was in line with the finding of Selvi et al. (2021) [23] who found TyG index a useful alternative to assess glycemic control. The lowest HbA1C levels were found among the subgroup of sufficient β-cell function/low TyG index, while the highest HbA1C levels were among the subgroup with insufficient β-cell function/high TyG index, indicating a positive impact of reducing IR and preserving β-cell function on glycemic control.

The result of this study showed no correlation between FCPGR and TyG index. This indicates that the presence of IR is independent of β-cell dysfunction and there is a variable degree of IR and β-cell dysfunction among the study patients. This finding agrees with the genetic role of β-cell dysfunction in T2DM pathogenesis [24]. The duration of T2DM was also not associated with TyG index. If recall bias was excluded, this finding could be explained by the IR that was already present before T2DM onset [25].

The frequency of hypertension among the recruited participants was 82 (82%), in addition there were significantly lower mean of FCPGR and significantly higher mean of TyG index among hypertensive patients in comparison with normotensives. These findings agree with IR as a common pathogenesis for both T2DM and hypertension and point to the importance of reducing IR in hypertensive patients [26].

The study results found an insignificant association between TyG index and treatment regimen except for pioglitazone and SGLT2i which were associated with low TyG index. These findings were in line with the mechanism of action of pioglitazone [27] and the finding of Waseda et al. (2018) who found that SGLT2i-treated patients had significantly reduced Homeostatic Model Assessment for IR (HOMA-IR) (a marker of IR) [28]. Unexpectedly, insignificant differences in TyG index were seen among those who were treated with and without metformin. Metformin is a well-known insulin sensitizer that reduces IR [29]. More research is needed to explain this result. However, the study of Pau et al. (2014) found no decrease in IR among patients with polycystic ovary syndrome patients after metformin treatment [30].

The glycemic status is the net result of interaction between β-cell function and IR; hyperglycemia indicates β-cell dysfunction and/or IR [23]. Our finding of significant correlation between both FCPGR and TyG index with HbA1C supports the potential of these markers as indicators of β-cell dysfunction and IR respectively, which subsequently can guide treatment transition to non-insulin therapy. In contrast, these markers were insignificantly correlated with BMI, duration of T2DM, duration and dose of insulin therapy making these variables alone unsuitable for deciding a management plan.

Limitations

This study had several limitations. This study used an FCPGR cutoff retrieved from Japanese T2DM patients, which may not fit the Iraqi population. Since higher cutoff can result in underestimation of β-cell function and may lead to unnecessary insulin treatment, while lower cutoff can overestimate β-cell function and impose the patients to the risk of hyperglycemia complications by unsuccessful transition to non-insulin therapy. Similarly, a TyG index cutoff value to describe IR in the Iraqi population is lacking, therefore this study used the most acceptable cutoff among available studies. Lastly, this study was conducted on a convenient sample, in which only patients who attend the Galiawa Diabetic and Endocrinology Teaching Center were enrolled in the study, which may limit the generalizability of the study.

## Conclusions

Both FCPGR and TyG index were well correlated with HbA1C. TyG index also significantly correlated with MetS index. Accordingly, the FCPGR and TyG index may provide good initial biomarkers for assessing β-cell dysfunction and IR respectively in T2DM on insulin therapy who cannot achieve glycemic control. 

Surprisingly, a significant number of T2DM patients on insulin therapy had preserved β-cell function (60, 60%), and almost all of them had high TyG index (95, 95%) indicating IR. These findings raised the question of whether these patients were receiving a proper treatment and whether they can be changed to non-insulin therapy.

Patients with preserved β-cell function and low TyG index were associated with better glycemic control. Knowledge about each individual patient's pathophysiological state can guide treatment choice through preserving β-cell function and targeting IR.
